# One-step synthesis of ZnO nanosheets: a blue-white fluorophore

**DOI:** 10.1186/1556-276X-7-470

**Published:** 2012-08-21

**Authors:** Sesha Vempati, Joy Mitra, Paul Dawson

**Affiliations:** 1 Centre for Nanostructured Media, School of Mathematics and Physics, Queen's University, Belfast, BT7 1NN, UK; 2 School of Physics, Indian Institute of Science Education and Research, Thiruvananthapuram, 695 016, India

**Keywords:** Zinc oxide, Nanosheets, Photoluminescence, Fluorophore

## Abstract

Zinc oxide is synthesised at low temperature (80°C) in nanosheet geometry using a substrate-free, single-step, wet-chemical method and is found to act as a blue-white fluorophore. Investigation by atomic force microscopy, electron microscopy, and X-ray diffraction confirms zinc oxide material of nanosheet morphology where the individual nanosheets are polycrystalline in nature with the crystalline structure being of wurtzite character. Raman spectroscopy indicates the presence of various defects, while photoluminescence measurements show intense green (centre wavelength approximately 515 nm) blue (approximately 450 nm), and less dominant red (approximately 640 nm) emissions due to a variety of vacancy and interstitial defects, mostly associated with surfaces or grain boundaries. The resulting colour coordinate on the CIE-1931 standard is (0.23, 0.33), demonstrating potential for use as a blue-white fluorescent coating in conjunction with ultraviolet emitting LEDs. Although the defects are often treated as draw-backs of ZnO, here we demonstrate useful broadband visible fluorescence properties in as-prepared ZnO.

## Background

There is a drive to replace conventional light sources with white-emitting sources based on light emitting devices (LEDs) on grounds of cost effectiveness and low power consumption. This route to white light emission involves various methodologies, for example, mixing the three primary colours (red, green and blue), the use of multilayer fluorescent structures or coating a yellow (partially transparent) fluorophore on to a blue-LED. This last approach is commonly employed where the LED is a blue-emitting GaN or InGaN device and the fluorophore is Ce:YAG. The coating is often referred to as a phosphor, although in fact the process is one of fluorescence; this is in contrast to ‘fluorescent’ lights where the coating is actually a phosphor or a mixture of several different phosphors comprised of transition metal or rare earth compounds. In addition to the spectral composition of the emitted light the other factor that needs to be considered in non-thermal light sources is that of efficiency; indeed, the efficiency is the main driving factor behind their development. This is often expressed in terms of lumens per Watt which is termed the efficacy of the source. In fact, this measure links spectrum and efficiency since the lumen is a measure that takes into account the spectral response of the human eye. Currently, in the UK, commercially available LED sources for domestic use are in the range of 55 to 70 lumens/W which contrasts with approximately 15 lumens/W for a tungsten-halogen bulb operating at approximately 3000 K.

In recent years, nanostructure-based, white-light emitting coatings have become a research area of general interest as a coating methodology for LEDs. These include ‘magic-sized’ CdSe [1] and a large variety of other materials in nano-particulate format, including CdSeS [2], Mn-doped ZnS [3], Mn-doped ZnSe [4], Ce^3+^-doped Y_3_Al_5_O_12_[5], doped YVO_4_:Ln^3+^, and silica-coated YVO_4_:Ln^3+^, where Ln^3+^ = Eu^3+^, Dy^3+^, or Tm^3+^[6] mixtures of rare earth phosphors [7] and rare earth-doped GdF_3_[8]. In addition, another class of coating materials currently attracting attention is that of wide band-gap semiconductors such as Ga_2_O_3_[9], In_2_O_3_[10], and ZnO. In particular, ZnO (band gap, 3.36 eV), which is the focus of this report, offers an interesting prospect, since various defects (e.g., oxygen vacancies (O_*v*_) and interstitials (O_*i*_), and zinc vacancies (*V*_Zn_) and interstitials (Zn_*i*_)) give rise to photoluminescence (PL) in different regions of the optical spectrum and, thus, yield the potential for a white light output. Much of the interest in the visible PL derives from the complexity of its origin where the specific origin of the various components has been a matter of some debate in the literature. Experimentally, there are many attempts to correlate specific components of the emission with the presence or deficiency of specific constituents, particularly oxygen, but it is often difficult to make definitive assignments. This situation is compounded by the fact that there is some variation in the calculation of the energies of deep trap levels associated with various defects, as exemplified in the overview band diagram in an article by Tam et al. [11]. Thus, even in very recent literature [12], the discussion of blue emission, for example, has been eschewed as a matter of rather indeterminate origin.

In this investigation, we report one-step, wet-chemical, low-temperature synthesis of pristine ZnO nanosheets (ZnO-NS) and their characterisation, principally with regard to PL. In addition to the requisite PL properties, it is important to have a straightforward, low-cost, and scalable fabrication protocol that could be applicable, for example, in the lighting industry or for fluorescent bio-labelling applications.

With regard to the PL, there are two important considerations. Firstly, in the literature, ZnO has been investigated for white-light emission in a number of different formats, for example, in doped form (using Cu^2+^[13] or Eu^3+^/Dy^3+^[14] dopants) or encapsulated in SiO_2_[15] or in the form of a polymer nanocomposite [16]. All of these approaches involve steps and/or materials in the fabrication process that are additional to the fabrication of the ZnO itself. However, as a broadband visible, fluorescent material, ZnO is less investigated in its pristine form. Thus, in contrast to previous studies, we do not use any dopants or host agents; instead, by taking advantage of various defects, we are able to demonstrate blue-white light emission from as-prepared ZnO nanostructures under exposure to ultraviolet light of energy greater than the bandgap. As an aside, we prefer to use the term fluorophores rather than phosphors since the PL does not involve long-lived (>1 ms) intermediate states.

Secondly, a key issue addressed here is the balance between the various visible emission components, encompassing consideration of their origin and the resultant colour composition as referenced to the CIE-1931 colour co-ordinate map. In effect the question is whether ZnO, stimulated by an UV source is spectrally viable as a white light source. While we do not purport to definitively disentangle the origin of the various, observed, visible PL components we do offer an interpretation with reference to some well-established previous observations and analysis in the field that can be put forward with some physical justification.

## Methods

All chemicals were used as received from Sigma-Aldrich Ltd (Gillingham UK) where the purity of the material is given in brackets. In a typical procedure, 50 mg of zinc acetate dihydrate (≥99.0%) is dissolved in 25 mL of methyl alcohol (MeOH ≥ 99.9%) with 25 mL of *p*-xylene (≥99.0%) subsequently added to this solution. In the discussion of the PL (‘Photoluminescence’ section), we comment in detail on the various trace species in the zinc acetate dihydrate. This mixture is refluxed at 80°C for 12 h in a 1-L round-bottom flask, giving rise to a white precipitate which is then washed thoroughly in MeOH and dried under vacuum. A previous study reports a very similar synthesis procedure with the same reactants [17] but at a lower temperature (60°C) and yields ZnO nanocrystals of an entirely different morphology from that reported here.

Physical characterisation in terms of microscopy was conducted on ZnO-NS supported on a holey carbon-coated TEM grid and consisted of micrograms from a scanning electron microscope (JEOL 6500 FESEM, JEOL Ltd., Akishima, Tokyo, Japan) and a transmission electron microscope (Philips Tecnai F20 FETEM, Philips Electronics, Amsterdam, Netherlands), as well as images from an atomic force microscope (Digital Instruments D3000 multi-mode SPM, Digital Instruments Corporation, CA, USA, now Bruker). In addition, X-ray diffraction (XRD) pattern was obtained using a Bruker D8 (Bruker AXS, WI, USA), 4-circle advanced X-ray diffractometer with Cu-Kα line, while Raman spectra were recorded using a laboratory-built set-up based on a Jobin-Yvon HR640 spectrometer (ISA Jobin Yvon, Longjumeau, France, now Horiba), where samples were illuminated with a 532-nm laser (Laserglow LLS-532, Laserglow Technologies, Ontario, Canada). Finally, the PL is investigated by illuminating the closed end of a quartz tube containing ZnO-NS, with a 355-nm laser. The PL spectrum is recorded with an Andor Technology SR-163 spectrometer equipped with an Andor DU-420 CCD camera (Andor Technology, Belfast, UK).

## Results and discussion

### Physical and structural characterisation

**Figure 1:**
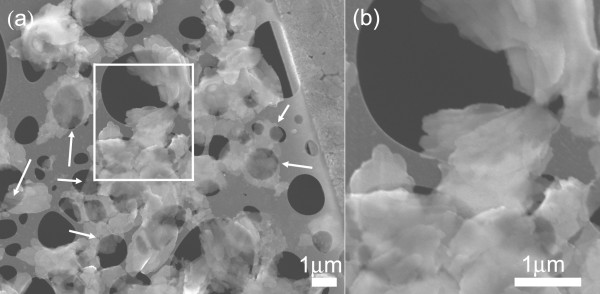
**SEM image of the ZnO nanosheets.** The arrows indicate regions of single to few nanosheets that are relatively electron transparent. Rectangular box in (a) is magnified and shown in (b).

**Figure 2:**
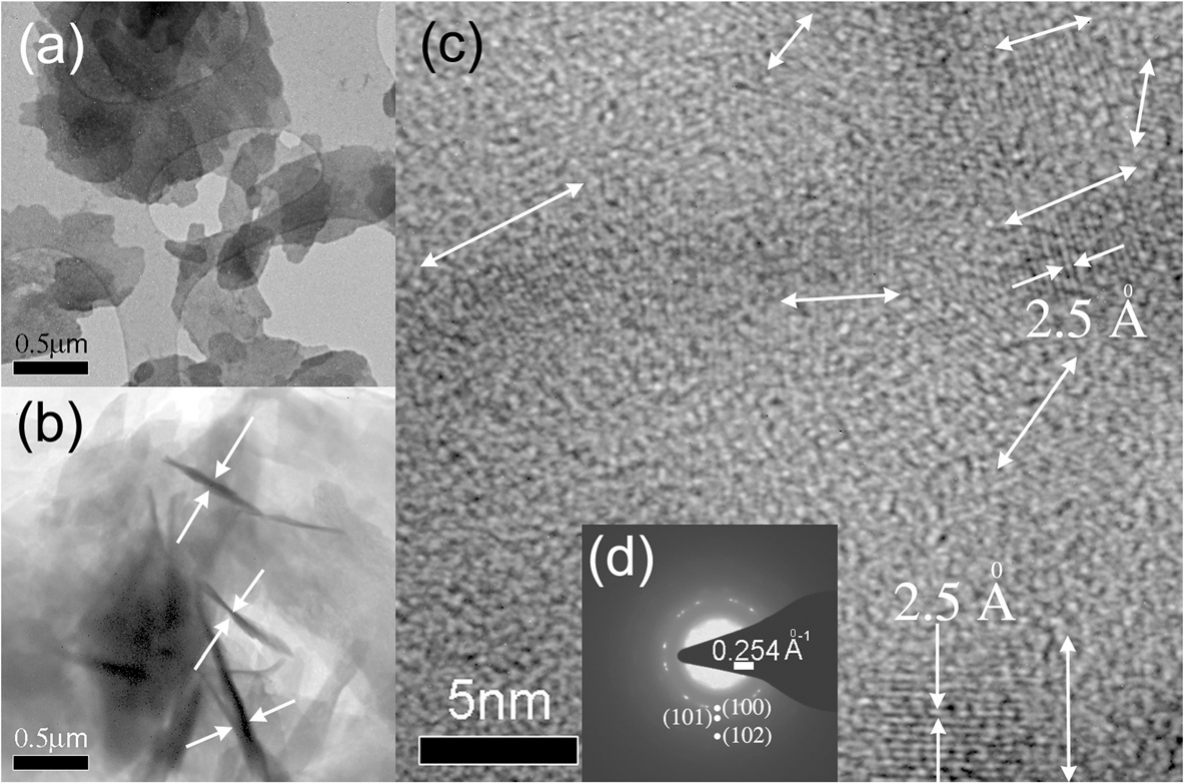
**TEM characterisation of ZnO-nanosheets.** (**a**) TEM image, (**b**) scanning transmission electron microscopy (STEM) image, (**c**) lattice resolved image with double-headed arrows indicating *c*-axis lattice growth direction, (**d**) SAED pattern.

**Figure 3:**
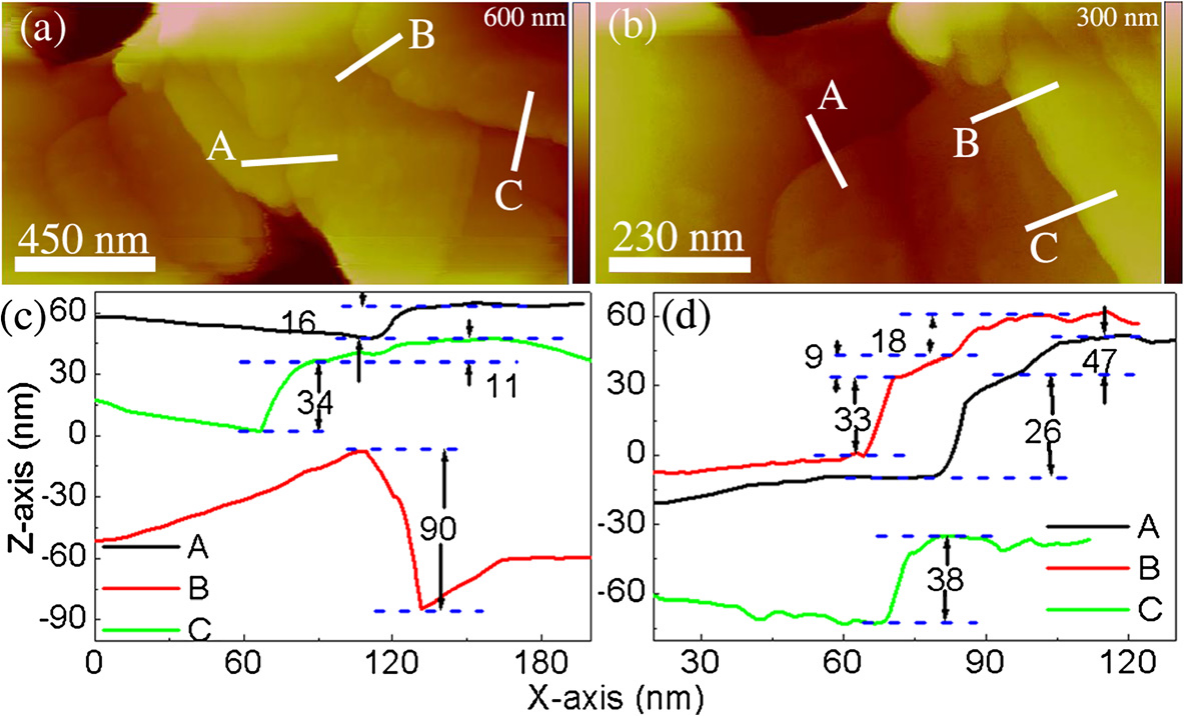
**AFM topographical images of regions with several ZnO-nanosheets (a) and (b).** Graphs (**c**) and (**d**) show line sections with height analysis taken from images (a) and (b), respectively. Images are coloured online.

The SEM data of Figure 1 illustrate a low-density material comprised of overlapping, thin sheets of ZnO where the individual sheets appear to have a lateral dimension on the micrometer scale. In certain regions (indicated by arrows), these structures appear quite transparent, even to the low energy (5 keV) beam employed for the SEM imaging. The TEM data are the most revealing, and a sample of such data is shown in Figure 2a,b,c along with a selected area electron diffraction (SAED) pattern (Figure 2d). The electron micrograms are complemented by the AFM data of Figure 3 which shows topographic images (Figure 3a,b) along with annotated AFM line sections, parts (c) and (d), corresponding to the images of (a) and (b), respectively. From the TEM and AFM results, there is further clear evidence of sheet morphology of ZnO with the sheets varying considerably in size, ranging from hundreds of square nanometres to several square microns. The TEM image, Figure 2a, illustrates regions of relatively uniform intensity bounded by distinct lines, as opposed to a more continuous grey-scale image; the discrete nature of these flat contrast regions suggests a sheet structure of ZnO of relatively uniform thickness. Interestingly, some sheets appear to be bent out of plane (Figure 2b) which conveniently facilitates an estimate of the sheet thickness to be in the range of a few tens of nanometres. This estimate is corroborated by the AFM topographic images of Figure 3a,b and the corresponding cross-sectional analysis (Figure 3c,d) which shows ZnO-NS thicknesses in the range of approximately 10 nm to 90 nm; however, it is possible that the thicker sheets may consist of several thinner sheets. No field flattening was applied to the AFM images prior to the thickness measurement.

Within a single NS, the HRTEM image of Figure 2c offers evidence of grains showing crystalline phases where the double-headed arrows indicate the *c*-axis lattice growth direction. The measured inter-planar spacing of 2.5 ± 0.06 Å is taken to be consistent with that for (002) planes reported in the literature, [18] thus indicating *c*-axis orientation in these regions. In fact, this spacing should be 2.6 Å that this value does not fall within the range of our measurement error may indicate a calibration error or material that is stressed, related to the fairly large proportion of amorphous-looking grain boundary material that seems to be present in this image.

In the SAED pattern of Figure 2d, only two diffraction planes, namely (100) and (101), are explicitly visible along with a faint indication of the (102) plane; other planes are not explicitly observed. Along with the earlier, lattice-resolved images, the SAED pattern suggest a polycrystalline phase for the ZnO-NS. In contrast to this case, single crystalline platelets have been reported in the literature [19].

**Figure 4:**
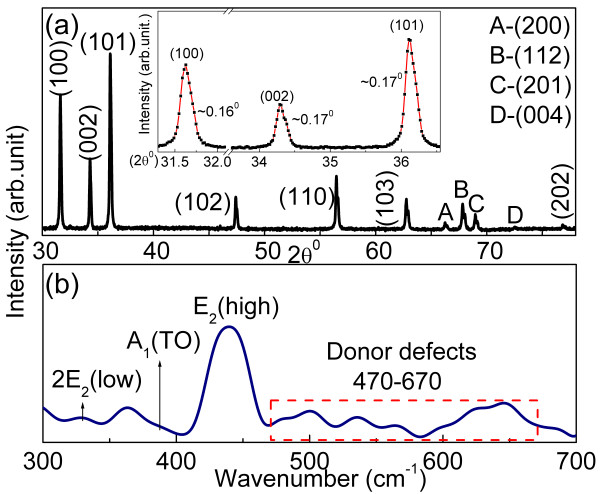
**XRD pattern and Raman spectrum from ZnO-NS.** (**a**) XRD pattern with inset showing FWHM of selected diffraction peaks in the range 30Â° to 38Â°. (**b**) Raman spectrum taken using a laser at 532 nm. Images are coloured online.

The XRD diffractogram of ZnO-NS is shown in Figure 4a. A high degree of crystallinity is indicated by the sharpness of the XRD peaks which exhibit a typical FWHM of approximately 0.16° to 0.17° (see inset to Figure 4a). The lattice parameters are calculated to be *a* = 3.266 Å and *c* = 5.229 Å; both the absolute values of these lattice constants and their ratio (*c/a* = 1.601) are consistent with a wurtzite crystal structure. The lack of diffraction peaks associated with impurities or reactants indicates that there is no significant contamination of the samples within the detection limits of XRD.

Finally, a Raman spectrum from ZnO-NS is shown in Figure 4b where peaks corresponding to E_2_ (high) at 439 cm^−1^ and 2E_2_ (low) at 329 cm^−1^ are annotated; overall, the spectrum is therefore consistent with a wurtzite-structured ZnO lattice. In addition, multiple peaks attributable to defects such as *V*_O_ and Zn_*i*_ can be seen spanning the range from 470 to 670 cm^−1^. Specifically, the peak at 545 cm^−1^, A_1_(LO), is known to be due to lattice oxygen vacancies. Further analysis of Raman spectra of ZnO has been given in our previous work addressing the synthesis of ZnO [20].

### Photoluminescence

Efficient visible photoluminescence in ZnO depends on the presence of defects such as *V*_O_ and Zn_*i*_ which may be bulk- or surface-related in nature. The latter is not necessarily limited to the ‘geometrical’ surface but may be more broadly classed as surface- or interface-induced defects to accommodate, for example, the effect of depletion regions at grain boundaries. Since the ZnO-NS here have a high surface area to volume ratio, it may be expected that surface defects play a significant role in the PL process. While defects of whichever type may not be detected directly with XRD, they can impart their signature to the Raman spectra but will be most manifestly evident in PL. The phenomenon of PL can thus provide both a useful means of characterisation of defects in ZnO and a pathway to exploitation as fluorescent coatings and markers.

**Figure 5:**
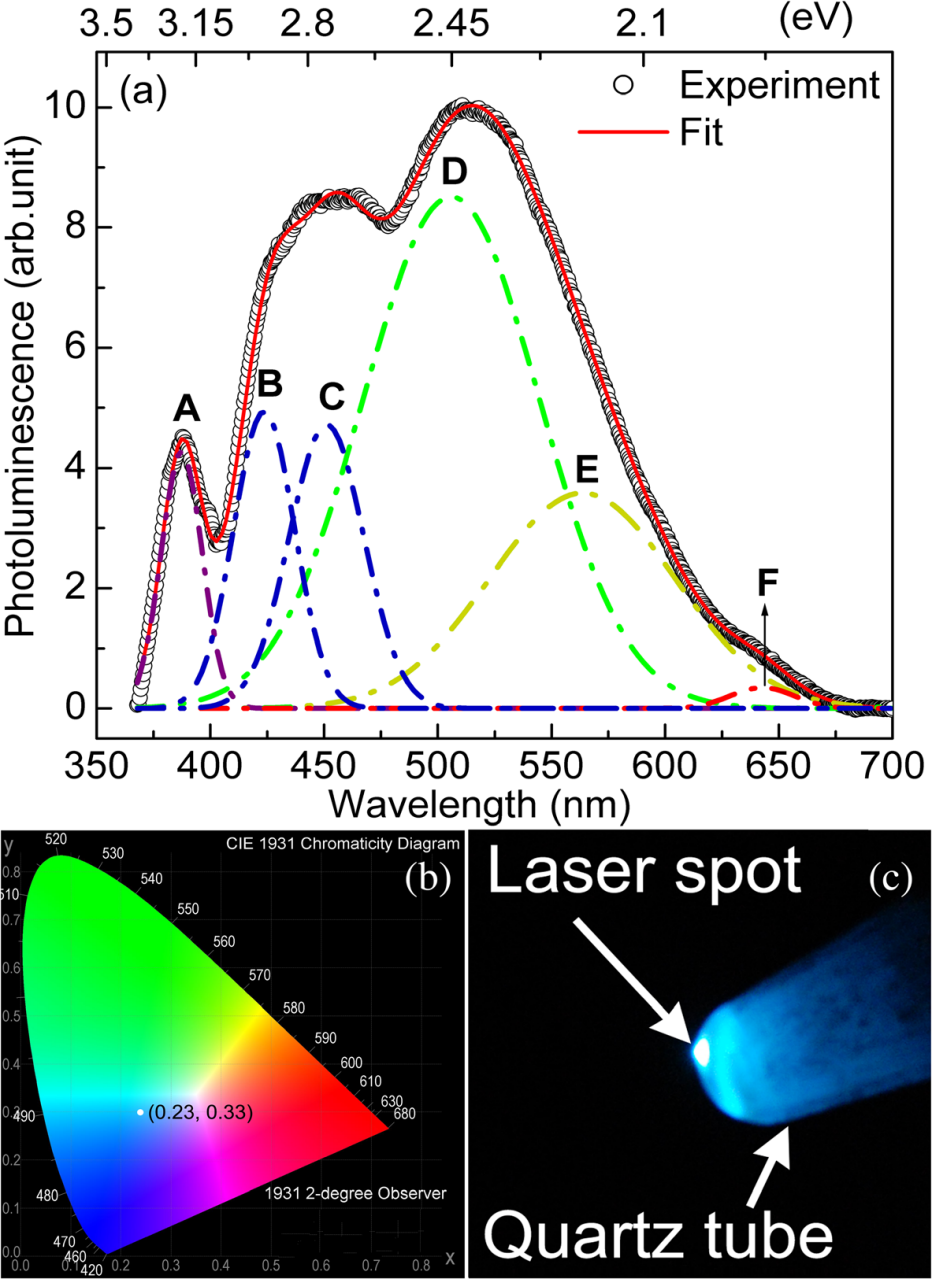
**Photoluminescence data for ZnO-NS.** (**a**) PL spectrum with deconvoluted peaks, A through F. The centre wavelengths of the component peaks, half-widths, and relative intensities along with the uncertainties in each quantity are given in Table 1. (**b**) Chromaticity coordinate plotted on CIE-1931 diagram. (**c**) Digital photograph of ZnO-NS in a quartz tube under laser illumination at 355 nm. Images are coloured online.

The PL response of the ZnO-NS is shown in Figure 5a with prominent visible bands centred at approximately 2.75 eV and 2.40 eV. To connect the luminescence properties to application as a fluorescent coating, the PL spectrum from the ZnO-NS is transposed to the CIE-1931 standard, yielding chromatic coordinate (0.23, 0.33) as shown in Figure 5b. The eye perception depicted by such chromatic coordinate (marked by a white dot in Figure 5b) is corroborated by the digital photograph of the PL shown in Figure 5c. The observed bluish-white colour matches that of Figure 5b, with the white spot in the middle being due to saturation of the camera where the input laser is most intense.

**Figure 6:**
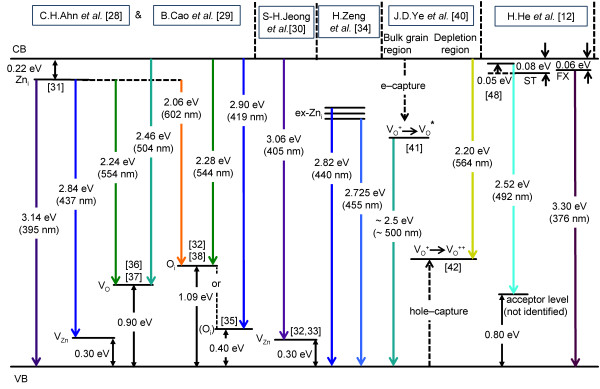
**Energy level diagram showing some of the principal defect levels in ZnO.** The ZnO has an assumed bandgap of 3.36 eV. Potential transitions between the various levels are colour coded and discussed in the text; the principal references taken for the basis of this figure are shown along the top with some additional feed-in references given within the figure in square brackets. Not shown are transitions in the red associated with either surface oxygen or surface OH groups. CB, conduction band; VB, valence band; FX, free exciton; ST, surface trap. Image is coloured online.

Attempting to trace the origin of the various colour components of the PL spectrum is not just of intrinsic interest but should assist in understanding how the ‘defect composition’ needs to be altered to effect a desired chromatic adjustment such as obtaining a truer white output. To this end, the PL spectrum has been deconvoluted into six Gaussian components in Figure 5a; the centre wavelength, intensities, and half-widths of the component peaks along with their respective uncertainties are given in Table 1. The composition of the spectrum is described with reference to Figure 6 which summarises the main PL transitions due to intrinsic defects as discussed in the literature. A further two possible, minor peaks may be accommodated in the spectral fit, and these will also be considered with reference to the six-fitted curve framework as well as the influence of extrinsic contaminants.

**Table 1:** **Centre wavelength, full-width-half-maximum (FWHM) and height of the deconvoluted PL peaks, A through F, of Figure**5a

**Peak**	**Centre**	**FWHM**	**Height**
	**(nm)**	**(nm)**	**(arb units)**
A	387.0±0.1	21.3±0.2	4.3±0.7
B	423.6±0.6	31.6±0.6	4.9±1.5
C	451.1±0.8	38.5±1.6	4.7±1.2
D	505.9±5.5	90.9±6.3	8.5±3.1
E	563.6±9.5	95.8±11.2	3.6±1.7
F	642.8±0.8	33.0±3.1	0.4±0.07

Firstly, and least controversially, is the peak in the ultraviolet (curve A) which is due to the recombination of excitons. This PL component, centred at 387 nm (approximately 3.20 eV), is intrinsic to all ZnO preparations and is, therefore, still clearly observed on samples that are virtually defect-free. Examples of such ZnO samples in nanosheet or platelet format [21,22] form a useful baseline in the sense of demonstrating that ZnO of very high crystallinity and near-perfect stoichiometry exhibits virtually no PL in the visible regime. A weak shoulder on the low energy (long wavelength) side of the experimental UV peak can be described by the insertion of a small additional peak centred in the range 395 to 399 nm (depending on the relative intensity used), with the effect that the main UV peak (curve A) is displaced by up to 0.04 to 3.84 eV. This value is closely comparable to the room temperature exciton peak energies of 3.23 and 3.26 eV reported by Chen et al. [22] and Cao et al. [23], respectively. This means that if the exciton peak is displaced by the generally accepted value of approximately 60 meV from the band edge, then the band gap lies approximately in the range 3.29 to 3.32 eV which is slightly less than the established bandgap for bulk ZnO of approximately 3.36 to 3.37 eV [24]. Such a value is not particularly anomalous in view of the variation in the band gap reported in the literature [25,26]. Also, a shift of the observed exciton peak to lower values could arguably be due to LO phonon replicas of the free exciton emission yielding a composite line shape with a peak value displaced downwards from the free exciton peak energy [27], though this line of argument may be rather more apt for highly crystalline samples. Here, however, the main focus of our interest is the visible PL. In order to reference the discussion of this PL to the literature, we will assume a bandgap of 3.36 eV, but need to bear in mind that some small adjustments in the various transition energies may be applicable to the ZnO-NS samples examined here.

In passing, we note that a possible candidate for the small additional peak just below 400 nm is the Zn_i_→ valance band (VB) transition (Figure 6) of energy 3.14 eV (395 nm). While Ahn et al. [28] report that this PL component disappears above 100 K (due to competition from the Zn_*i*_→*V*_Zn_ transition), Cao et al. [29] track its evolution to room temperature, although at somewhat diminished intensity. An alternative candidate is conduction band the (CB)→*V*_Zn_ transition at 3.06 eV (405 nm) that Jeong et al. [30] consider to account for a violet emission observed at a centre wavelength of 401 nm in their work.

Starting at the blue end of the visible spectrum, the balance of the evidence is that the PL is primarily associated with Zn defects. Much discussion of experimental data in the literature [28-30] is based on calculations of the energy level of Zn_*i*_ at 0.22 eV below the conduction band [31] and of *V*_Zn_ as an acceptor level 0.30 eV above the valance band [32,33]. A transition between these levels, i.e. Zn_*i*_→*V*_Zn_, would lead to emission centred at 2.84 eV (437 nm) and offers a possible origin of curve C of Figure 5a, though not in particularly good agreement. More recently, using a variety of stimulating wavelengths above and below the band gap, Zeng et al. [34] have given good grounds for assigning a PL emission peak at 415 nm to the Zn_*i*_→VB transition (with the implication that Zn_*i*_ is a deeper lying level at approximately 0.37 eV below the CB), while various extended Zn_*i*_ states give rise to emission in the 440- to 455-nm range, depending on annealing and other conditions; these wavelengths correspond reasonably well to those of curves B (423 nm) and C (451 nm) in Figure 5a. However, as a preview to consideration of oxygen-related defects, it is also interesting to note that an evaluation of O_*i*_ at only 0.4 eV above the VB [35] (rather than the more generally quoted value of approximately 1.09 eV [28]) leads to the possibility of a CB→O_*i*_ transition of 2.96 eV or 419 nm. Nonetheless, we consider that the weight of evidence for the blue emission lies with transitions from Zn_*i*_ states (or extended Zn_*i*_ states) to *V*_Zn_ states or VB, although our data do not permit us to discriminate between differences in these transitions reported in the literature.

We consider next the main mid-visible PL, i.e. primarily the ‘green’ component of the emission which is generally associated with oxygen-related defects in the literature. In Figure 5a, the main contribution to this mid-visible emission is a component centred at 506 nm (curve D) with a smaller, but still significant contribution at 564 nm (curve E) which we consider in tandem; the minor red component at 643 nm (curve F) is addressed separately. Based on calculation of the *V*_O_ level at 0.9 eV above the VB [36] and the electron paramagnetic resonance experiments of Vlasenko et al. [37], Ahn et al. [28] and Cao et al. [29] identify two contributions to the green emission at 2.24 eV (554 nm) and 2.46 eV (504 nm) due to Zn_*i*_→*V*_O_ and CB→*V*_O_ transitions, respectively. It has also been suggested [28,29] that PL at 2.28 eV (544 nm) will be generated by a CB→O_*i*_ transition (assuming that the O_*i*_ level is 1.09 eV above the VB; see Ahn et al. [28] and Li et al. [38] and references therein), as shown in Figure 6. In addition, a transition from the CB to an oxygen antisite defect, O_Zn_, has been held responsible for PL at 2.38 eV (521 nm) [32]. While the centre wavelength of the CB→*V*_O_ transition (504 nm) is in good agreement with curve D of Figure 5a (506 nm), those for the Zn_*i*_→*V*_O_, CB→O_*i*_ or CB→O_Zn_ transitions are in progressively worse agreement with respect to curve E. We, therefore, prefer an interpretation of the mid-visible PL which we have found very useful in our previous work on ZnO films and nanostructures [20,39], namely that of Ye et al. [40]. This, in turn, is based on the investigations of Vanheusden et al. [41] and van Dijken et al. [42]. The former [41] suggests a non-radiative electron capture from the CB by a singly charged oxygen vacancy (*V*_O_^+^) leading to an unstable state that recombines with a photoexcited hole in the VB, yielding PL at approximately 2.45 to 2.50 eV (approximately 506 to 496 nm); this process is associated with bulk material. Importantly, this interpretation also argues that grain boundary-induced depletion regions lead to the formation of a deeply trapped, doubly charged oxygen vacancy (*V*_O_^++^) state which undergoes radiative recombination with a CB electron to yield PL of energy approximately 2.20 eV (564 nm), which is in very good agreement with curve E of Figure 5a. In this picture, the relative PL contributions at the two wavelengths, thus, depend on the ratio of bulk to depletion (typically 5- to 10-nm deep) volumes which is clearly a function of the ZnO structure and morphology [40]. In summary, applying the interpretation of Ye et al. [40] yields very good agreement of both transition wavelengths with experimentally derived centre wavelengths, where the greater contribution to the overall emission spectral profile originates from bulk material, but with a significant surface contribution.

Before leaving the green emission, we note that in experiments on the temperature dependence of PL from ZnO platelet structures [12], the band at approximately 2.5 eV exhibited negative thermal quenching, i.e. increasing intensity with increasing temperature. This was attributed to the thermal activation of electrons from a surface trap state (80 meV below the CB) to a shallow state 50 meV below the CB with subsequent decay to a deep trap level 0.8 eV above the VB, as shown in Figure 6. While the various states involved were not chemically specified, there is, in common with the model of Ye et al. [40], the fact that the process originates with (non-radiative) transitions at or near the CB edge prior to the transition responsible for the observed PL. Indeed, relevant to both pathways discussed above, it is interesting to suggest a link to a placement of the *V*_O_ level at only 50 meV below the CB [43], tying in with the shallow donor level of He et al. [12] and the precursor state to the formation of the *V*_O_^+^ state discussed by Ye et al. [40] and Vanheusden et al. [41]. In terms of the lower state for the transition from this neutral *V*_O_ level, He et al. [12] simply suggest an unspecified deep acceptor level. However, from the above discussion, it is manifestly clear that a serious problem in attributing peaks in PL to oxygen defect states is the wide disagreement in the energy levels attributed to these states. However, in concluding this analysis of the main green PL components, we reiterate the interpretation of Ye et al. [40] which we have applied consistently in our previous analysis of ZnO samples and devices [20,39].

Moving to longer wavelengths, Figure 5a shows a weak red output (curve F at 643 nm). Compared to other PL components, the red component has received relatively little focused attention in the literature; yet, it is the enhancement of this component from ZnO that is needed, both generally and in the work reported here, to produce a better white colour balance for PL display applications. From the long-standing work of Studenikin et al. [44], at room-temperature, the red PL has generally been associated with excess (surface) oxygen; this conclusion was based on various annealing studies in oxygen and forming gas atmospheres. No upper and lower states for the transition were specified, but Tam et al. [11] have subsequently suggested a defect complex, e.g. *V*_O_Zn_*i*_ as the lower state in red/yellow PL. More recently, though, a systematic shift of the red emission wavelength with the UV band edge absorption (dependent on different ZnO thin film preparations) led Marotti et al. [45] to suggest a radiative transition from an upper state that is referenced to the conduction band, possibly Zn_*i*_, to a deep level where *V*_O_ is again suggested. However, in their work, this process does not seem to persist above approximately 200 K, and so we are left with the link to surface oxygen to account for the red emission. This red component could be boosted by annealing in oxygen, facilitating a greater density of surface oxygen, possibly at the expense of OH surface moieties (see below). Interestingly, a good red PL yield (and thus a better white output) has recently been achieved with more complex ZnO-silicon nanocomposite structures [46] where appeal is made to the ZnO grain-Si interface region, specifically to Zn-Si-O linkages and thence to the eventual formation of ZnSiO_4_, to explain spectral peaks centred at 420 and 640 nm, respectively. However, these peak wavelengths are almost exactly coincident with two of the peaks observed in the ZnO-NS here and may, therefore, be subject to characterisation in terms of only the ZnO material itself.

As a final point in the spectral deconvolution, we note that it is possible to include a small component in the region of 580 nm. The motivation to include such a component is a possible connection to OH surface states. A radiative decay path involving a transition from OH states to deep-lying states (or possibly the VB edge) with emission centred at 576 nm (approximately 2.15 eV) was posited by Tam et al. [11] and developed by Djurisic et al. [47] in the context of examining PL from ZnO prepared by a low-temperature, hydrothermal technique where stable OH species may be supported at the surface of the ZnO, generally in the form of Zn(OH)_2_[48]. In common with that fabrication technique where the maximum temperature reached was 90°C, the technique used here proceeds at low temperature with a reflux cycle at only 80°C and also involves a hydrated form of zinc acetate as one of the starting materials. Thus, if a weak yellow-orange component is included in the spectral analysis, we attribute its existence to transitions involving surface OH groups; this appears more physically viable than the more bulk-related Zn_*i*_→O_*i*_ emission at 602 nm (Figure 6) which is unaffected by surface modification [38].

Finally, we consider the presence of various contaminants or extrinsic defects in ZnO and whether these might account for some of the visible PL. With a cost benefit factor of > 60 in using puriss grade zinc acetate dihydrate (99%) as opposed to trace metal basis version (99.99%), there comes the issue of the inclusion of a variety of cationic contaminants or dopants mostly at the < 5 mg/kg level; these include the ferromagnetic elements Fe, Co, and Ni commonly used in deliberate doping of Zn to induce a magnetic response in the material, as well as Cs, Cr, Cu, Mg, Mn, and Pb (Na and K are also present at levels up to 50 mg/kg). These levels correspond to the range approximately 7 to 40 ppm depending on atomic mass (and up to approximately 400 ppm for the case of Na). The Additional file 1 gives a survey of the role of these various contaminants with references [49-77] numbered contiguously with the main text. The salient features from this survey are as follows: In very many cases, the doping level used is very high indeed by normal semiconductor standards, ranging from 1,000 ppm upwards but more typically in the 1% to 10% range and sometimes even higher. Thus, any observable effects on the PL attributable to extrinsic doping need to be scaled back very significantly when applied to the samples considered in this work. Secondly, in the bulk of the studies considered, the effect of extrinsic doping is to attenuate or even quench the main PL bands in the visible, particularly the green peak. Finally, it is noticeable that the vast majority of explanations tendered for changes in PL upon doping appeal to the effect that the extrinsic dopants have on transitions involving the native defects (Zn_*i*_, *V*_O_, etc.); this line of argument generally includes the few instances in which enhanced peaks in the PL are reported. In the exceptional cases where a new PL peak is causally linked with a transition involving a level associated directly with the presence of the dopant species in the host lattice, the effect is weak and therefore observed against a benign background in which other significant visible PL contributions are absent. In concluding this section, the weight of evidence is very much that the bulk of the visible PL is due to intrinsic rather than extrinsic defects.

## Conclusion

A one-step, wet-chemical synthesis technique has been implemented to obtain polycrystalline ZnO-NS with the potential for application as a blue-white fluorescent coating for UV sources. The integrity of the material is established using electron and atomic force microscopies, along with XRD, with the various defects first showing their fingerprint in Raman spectroscopy analysis. In addition to the benchmark UV near-band-edge or excitonic emission, UV-illuminated ZnO-NS emit visible PL, attributable to intrinsic rather than extrinsic defects, that extends right across the visible spectrum. This emission appears white with a blue tint with colour coordinate (0.23, 0.33) with a relatively complex component spectrum. A broadly based analysis of the PL output suggests that the bulk of the output at the blue end of the spectrum, specifically peaks centred at 424 and 451 nm, is due to transitions from (extended) interstitial zinc defect states to zinc vacancy states or the VB. The main output in the mid-visible range is comprised of two components at approximately 506 and 564 nm, and is attributed to transitions involving oxygen defects, specifically the bulk-related *V*_O_^+^→VB transition for the 506-nm emission and depletion region-related CB→*V*_O_^++^ transition for the 564-nm emission. Evidence is considered that indicates that surface oxygen is connected with the red emission peak at 643 nm, while the possibility is flagged of a small yellow-orange component at approximately 580 nm connected with the presence of OH surface groups. It is these long wavelength components, particularly the red component, that require boosting in order to yield a more balanced white output.

## Competing interests

The authors declare that they have no competing interests.

## Authors' contributions

SV was responsible for carrying out the experimental work and the basic results analysis, as well as drafting the manuscript. JM helped design the experiment and assisted with results analysis. PD instigated and gave overall supervision to the project, in addition to substantive editing of the manuscript. All authors read and approved the final manuscript.

## Supplementary Material

Additional file 1 This document gives a survey of the role of various contaminants in PL from ZnO. Click here for file
