# Supplementary material for: One-step synthesis of ZnO nanosheets: a blue-white fluorophore
Source: Nanoscale Res Lett. 2012 Aug 21;7(1):470. doi: 10.1186/1556-276X-7-470 (PMC3522028; doi:10.1186/1556-276X-7-470)
Supplement: Additional file 1 — This document gives a survey of the role of various contaminants in PL from ZnO. [file 1556-276X-7-470-S1.doc]

**Additional Documentation**

In the main text it was noted that there is a large variety of contaminant metallic species that are presumably present in the ZnO-NSs due to the use of puriss grade zinc acetate dihydrate in the preparation procedure. To repeat the catalogue these are Fe, Co, Ni, Cs, Cr, Cu, Mg, Mn and Pb, all present at the < 5 mg/kg level, and Na and K at the < 50 mg/kg level. Of these Cu is almost universally present in ZnO preparations and is known to lead to green emission (peak centred at 500-510 nm) where the examination of such emission traces back to the early work of Dingle [49]; it has been further studied in some detail, e.g. as a function of temperature and pressure. [50] However, to place it in context, the unequivocal observation of green emission due to Cu^+^ or Cu^2+^ present at the few ppm level [49, 51] is accompanied by exciton emission that is greater in intensity by one or two orders of magnitude or even more. Moreover, when Cu doping levels are sufficiently high (0.08%-4%) to have a significant impact at the higher end of the green PL intensity scale the effect is to quench exciton emission by comparison to the case of otherwise equivalent undoped samples. [52] With the presumed presence of Cu in our samples on the few10’s ppm scale, it is certainly consistent that the exciton emission remains significant while, from the literature, it is clear that Cu-induced PL can account for only a very small part of the green PL. We consider next the effect on PL of doping ZnO by the ferromagnetic elements, Fe [53-55]; Ni [56-58]; and Co [59-61]. In all cases cited, the doping regime examined vastly exceeds the 10 ppm level, extending up to the order of 10%, but the effect is simply to modify the PL that is already present on equivalent undoped samples due to intrinsic defects. Indeed, in most cases the effect of the dopant is to significantly attenuate the visible PL. Even where a blue emission appears (while the green emission is quenched) upon increased Fe-doping, [54] the analysis and interpretation is largely in terms of a Fe-induced alteration of intrinsic defect transitions. Similar trends emerge in doping with other elements. In a study of granular ZnO films with 10% Cr doping there is relatively little effect on the PL with respect to the undoped case. [62] Where changes in the visible PL with (2%) Cr doping have been observed, such as a broadening and/or an intensity change [63, 64], they are unambiguously attributed to the effect the doping (or the annealing of doped samples) has on the density of and/or the transition probability associated with intrinsic defects underpinning the PL. Likewise, an increase in non-radiative recombination processes with increasing Mn concentration is considered to be responsible for the decrease [65] or even quenching [66] of the PL intensity due to intrinsic defects in Mn-doped ZnO. The most concentrated of the accidental dopants (at up to 50 mg/kg of zinc acetate dihydrate) are K [67, 68]; and Na [69, 70]. Xu *et al.* [67] depart from the bulk of the literature in that they positively attribute a blue peak at 470 nm to K-interstitials (rather than the effect of the dopant on intrinsic defects); however, the emission, observed against an almost null background of other PL, is an order of magnitude less that the UV exciton emission and is associated with a doping level of 1 at%. Another ‘exception’ is the rare case of Pb-doped ZnO [71] although the green emission is quite markedly enhanced with Pb doping, interpolation back to the few ppm doping level would not account any significant visible PL in our case. For the remaining contaminant species the reader can confirm consistent observations (modest changes or significant attenuation of visible PL, but only at very high doping levels, explained in terms of the effect on dopants on intrinsic defect emission) for the cases of Ca-doped ZnO [72] Cd-doped ZnO [73, 74]; and Mg-doped ZnO [75, 76] where ref. [77] also examines (Al, Li)- co-doped ZnO thin films.

49. Dingle R: **Luminescent Transitions Associated With Divalent Copper Impurities and the Green Emission from Semiconducting Zinc Oxide.** *Phys Rev Lett* 1969, **23:**579.

50. Su FH, Liu YF, Chen W, Wang WJ, Ding K, Li GH, Joly AG, McCready DE: **Temperature and pressure dependences of the copper-related green emission in ZnO microrods.** *J Appl Phys* 2006, **100:**013107.

51. Garces NY, Wang L, Bai L, Giles NC, Halliburton LE, Cantwell G: **Role of copper in the green luminescence from ZnO crystals.** *ApplPhysLett* 2002, **81:**622.

52. Ghosh T, Dutta M, Mridha S, Basak D: **Effect of Cu Doping in the Structural, Electrical, Optical, and Optoelectronic Properties of Sol- Gel ZnO Thin Films.** *J Electrochem Soc* 2009, **156:**H285.

53. Chen AJ, Wu XM, Sha ZD, Zhuge LJ, Meng YD: **Structure and photoluminescence properties of Fe-doped ZnO thin films.** *J Phys D: Appl Phys* 2006, **39:**4762.

54. Srivastava AK, Deepa M, Bahadur N, Goyat MS: **Influence of Fe doping on nanostructures and photoluminescence of sol–gel derived ZnO.** *MaterChemPhy* 2009, **114:**194.

55. Panigrahy B, Aslam M, Bahadur D: **Effect of Fe doping concentration on optical and magnetic properties of ZnO nanorods.** *Nanotechnology* 2012, **23:**115601.

56. He JH, Lao CS, Chen LJ, Davidovic D, Wang ZL: **Large-Scale Ni-Doped ZnO Nanowire Arrays and Electrical and Optical Properties.** *J Am Chem Soc* 2005, **127:**16376.

57. Iqbal J, Wang B, Liu X, Yu D, He B, Yu R: **Oxygen-vacancy-induced green emission and room-temperature ferromagnetism in Ni-doped ZnO nanorods** *New J Phys* 2009, **11:**063009.

58. Liu Y, Wang T, Sun X, Fang Q, Lv Q, Song X, Sun Z: **Structural and photoluminescent properties of Ni doped ZnO nanorod arrays prepared by hydrothermal method** *Appl Surf Sci* 2011, **257:**6540.

59. Vempati S, Shetty A, Dawson P, Nanda K, Krupanidhi SB: **Cobalt-doped ZnO nanowires on quartz: Synthesis by simple chemical method and characterization.** *J Cryst Growth* 2012, **343:**7-12.

60. Hays J, Reddy KM, Graces NY, Engelhard MH, Shutthanandan V, Luo M, Xu C, Giles NC, Wang C, Thevuthasan S, Punnoose A: **Effect of Co doping on the structural, optical and magnetic properties of ZnO nanoparticles.** *J Phys: Condens Matter* 2007, **19:**266203.

61. Chang YQ, Wang PW, Ni SL, Long Y, Li XD: **Influence of Co Content on Raman and Photoluminescence Spectra of Co Doped ZnO Nanowires.** *J Mater Sci Technol* 2012, **28:**313.

62. Hu YM, Chen YT, Zhong ZX, Yu CC, Chen GJ, Huang PZ, Chou WY, Chang J, Wang CR: **The morphology and optical properties of Cr-doped ZnO films grown using the magnetron co- sputtering method** *Appl Surf Sci* 2008, **254:**3873.

63. Wang B, Iqbal J, Shan X, Huang G, Fu H, Yu R, Yu D: **Effects of Cr-doping on the photoluminescence and ferromagnetism at room temperature in ZnO nanomaterials prepared by soft chemistry route.** *MaterChemPhy* 2009, **113:**103.

64. Fu CF, Han LF, Liu C: **Effects of annealing on the structural and photoluminescence properties of Zn0.98Cr0.02O thin films prepared by magnetron sputtering.** *Phys Status Solidi A* 2011, **11:**2661.

65. Maiti UN, Ghosh PK, Nandy S, Chattopadhyay KK: **Effect of Mn doping on the optical and structural properties of ZnO nano/micro-fibrous thin film synthesized by sol–gel technique.** *Physica B* 2007, **387:**103.

66. Zhang XT, Liu YC, Zhang JY, Lu YM, Shen DZ, Fan XW, Kong XG: **Structure and photoluminescence of Mn-passivated nanocrystalline ZnO thin films** *J Cryst Growth* 2003, **254:**80.

67. Xu L, Gu F, Su J, Chen Y, Li X, Wang X: **The evolution behavior of structures and photoluminescence of K-doped ZnO thin films under different annealing temperatures.** *J Alloy Compd* 2011, **509:**2942.

68. Gupta MK, Sinha N, Kumar B: **p-type K-doped ZnO nanorods for optoelectronic applications.** *J Appl Phys* 2011, **109:**083532.

69. Lai J-J, Lin Y-J, Chen Y-H, Chang H-C, Liu C-J, Zou Y-Y, Shih Y-T, Wang M-C: **Effects of Na content on the luminescence behavior, conduction type, and crystal structure of Na-doped ZnO films** *J Appl Phys* 2011, **110:**013704.

70. Lü J, Huang K, Zhu J, Chen X, Song X, Sun Z: **Preparation and characterization of Na-doped ZnO thin films by sol–gel method.** *Physica B* 2010, **405:**3167.

71. Ahmad M, Pan C, Yan W, Zhu J: **Effect of Pb-doping on the morphology, structural and optical properties of ZnO nanowires synthesized via modified thermal evaporation** *Mat Sci Eng B-Solid* 2010, **174:**55.

72. Karthikeyan B, Pandiyarajan T, Mangaiyarkarasi K: **Optical properties of sol–gel synthesized calcium doped ZnO nanostructures.** *Spectrochim Acta A* 2011, **82:**97.

73. Liu JZ, Yan PX, Yue GH, Chang JB, Zhuo RF, Qu DM: **Controllable synthesis of undoped/Cd-doped ZnO nanostructures.** *Mater Lett* 2006, **60:**3122.

74. Ogawa Y, Fujihara S: **Blue Luminescence of MgZnO and CdZnO Films Deposited at Low Temperatures.** *J Electrochem Soc* 2007, **154:**J283.

75. Shi Q, Zhang J, Zhang D, Wang C, Yang B, Zhang B, Wang W: **Red luminescent and structural properties of Mg-doped ZnO phosphors prepared by sol–gel method.** *Mat Sci Eng B-Solid* 2012, **177:**689.

76. Fujihara S, Ogawa Y, Kasai A: **Tunable Visible Photoluminescence from ZnO Thin Films through Mg-Doping and Annealing.** *Chem Mater* 2004, **16:**2965.

77. Fujihara S, Naito H, Kimura T: **Visible photoluminescence of ZnO nanoparticles dispersed in highly transparent MgF2 thin-films via sol-gel process.** *Thin Solid Films* 2001, **389:**227-232.
